# Negative Consequences on the Growth, Morphometry, and Community Structure of the Kelp *Macrocystis pyrifera* (Phaeophyceae, Ochrophyta) by a Short Pollution Pulse of Heavy Metals and PAHs

**DOI:** 10.3390/toxics9080190

**Published:** 2021-08-18

**Authors:** Roddy Jara-Yáñez, Andrés Meynard, Gladys Acosta, Nicolás Latorre-Padilla, Carolina Oyarzo-Miranda, Francisco Castañeda, Florentina Piña, Jorge Rivas, Cristian Bulboa, Loretto Contreras-Porcia

**Affiliations:** 1 Departamento de Ecología y Biodiversidad, Facultad de Ciencias de la Vida, Universidad Andres Bello, Santiago 8370251, Chile; roddy.jara@gmail.com (R.J.-Y.); meynardster@gmail.com (A.M.); gladyszoo@yahoo.es (G.A.); nlatorrepadilla@gmail.com (N.L.-P.); karo.oyarzo@gmail.com (C.O.-M.); fra.castaneda@gmail.com (F.C.); florentinapina1996@gmail.com (F.P.); jrivasperez@gmail.com (J.R.); 2 Centro de Investigación Marina de Quintay (CIMARQ), Facultad de Ciencias de la Vida, Universidad Andres Bello, Valparaiso, Quintay 2531015, Chile; 3 Center of Applied Ecology and Sustainability (CAPES), Santiago 8331150, Chile; 4 Instituto Milenio en Socio-Ecología Costera (SECOS), Santiago 8370251, Chile

**Keywords:** community structure, ecosystem health, growth, heavy metals, *Macrocystis pyrifera*, marine contamination, morphometry, polycyclic aromatic hydrocarbons (PAHs)

## Abstract

The study of pollution effects in the marine environment has become important in recent decades, and the exposure to simultaneous pollutants has become especially relevant. Indeed, the study of key organisms, such as ecosystem engineers, can show a broader view of the effects of pollutants. In this context, we evaluate in situ the effects of a short (7-day) pollution pulse of combined solutions of heavy metals and polycyclic aromatic hydrocarbons (PAHs) (Cu + PAHs, Cd + PAHs, Cu + Cd, and Cu + Cd + PAHs) on the development and morphological features of *Macrocystis pyrifera* sporophytes over a period of 90 days. Additionally, we determined the effects on the community structure associated with this kelp. This study evidenced a smaller number of blades and a decreased size of blades and holdfasts, as well as the death of individuals exposed to a secondary mix of metals (Cu + Cd) and a tertiary mix of pollutants (Cu + Cd + PAHs). Regarding the effects on the accompanying fauna, low richness and diversity were registered. *M. pyrifera* grazers, according to the mixture of pollutants, were either absent or diminished. These results show that the pulse of contamination in the early stages of *M. pyrifera* negatively affects its development and morphometry, as well as its role as an ecosystem engineer, due to a negative alteration in the species composition.

## 1. Introduction

Coastal areas are particularly important, due to the high abundance and diversity of marine life. However, coastal zones all over the world have become extensively degraded by growing human occupation and an increase in toxic chemical discharges, which in turn negatively affects biodiversity and ecosystem services, among other effects [1]. Therefore, it is imperative to estimate the distribution patterns of pollutants in coastal regions and their potential impacts on marine ecosystems.

Throughout the Chilean coastline, high levels of heavy metals and polycyclic aromatic hydrocarbons (PAHs) have been found in seawater, sediments, and marine organisms [2,3,4,5,6,7]. Heavy metals are inorganic naturally occurring compounds in the marine environment, but their concentrations have increased significantly because of anthropogenic intervention [1,8,9]. Historically, heavy metals have been one of the most common pollutants in the Chilean shores, originating primarily from mining and industrial activities, as well as from other land-based discharges and atmospheric deposition. On the other hand, PAHs are persistent organic compounds found in coastal areas mainly because of human activities, such as land-based discharges, deposition, ship accidents, and, more frequently since the 1900s, the rise in oil spill events [6,10,11,12,13]. Given their mutagenic and carcinogenic properties, which cause significant risks to aquatic life [13], PAHs were included in the priority lists of pollutants of the European Union (EU) and the United States Environmental Protection Agency (EPA) and are currently on the hazardous substance lists provided by the Agency for Toxic Substances and Disease Registry (ATSDR). 

Several authors have reported negative effects of heavy metals and PAHs at different biological levels in the marine environment, but these studies have mainly assessed the toxic effects of individual pollutants [14,15,16,17]. Nonetheless, the synergistic effects of heavy metals and PAHs have been recently documented [18,19,20], which is extremely worrying since both pollutants are frequently co-occurring in environmental matrices worldwide. The enrichment of coastal waters with chemical mixtures could increase, for example, the individual toxicity of heavy metals, and affect not only single species but also the diversity and structure of marine communities through cascading trophic consequences (bottom-up and top-down effects). However, it is known that the effects of pollutants are species- and even population-specific [16,17,18,19,20]. 

Foundation species are key taxa that increase food-web complexity and richness by creating habitats, influencing physical conditions, and influencing ecosystem processes [21,22,23,24,25,26,27,28,29]. Negative effects on foundation species (e.g., on their development, growth, and changes in physiological or morphological traits) impact multiple levels of biological organization, causing a reduction in biodiversity and disruptions in community structure and ecosystem services [7,30]. *Macrocystis pyrifera* (Linnaeus) C. Agardh has been described as a foundation species in subtidal coastal marine ecosystems worldwide [26,27]; it offers different habitats for various algae, invertebrate, and vertebrate species, and is an important food source for many grazers. Moreover, its biomass is directly related with species richness. Sporophytes of *M. pyrifera*, which correspond to the diploid macroscopic phase of the life cycle of this species, are highly tolerant to heavy metal exposure [30], and have been considered as biomonitors in field studies for heavy metal pollution due to their great capacity for bioaccumulation [31,32].

Despite the ecosystem importance of *M. pyrifera* and the negative effects caused by the exposure to high levels of heavy metals and PAHs in marine organisms, no studies have examined both the individual and combined effects of these toxics, nor their effects on its associated fauna/flora and community structure. In this context, the first objective of this study was to examine, in vitro and in field conditions, the direct effects of exposure to combined solutions of heavy metals and PAHs on the growth and morphology of juvenile sporophytes of *M. pyrifera*. The second objective was to study the growth of these juvenile individuals under field conditions and assess the indirect effects of toxics on its associated fauna/flora through community structure analyses.

## 2. Materials and Methods

### 2.1. Exposure of M. pyrifera to Treatment

A total of 250 juvenile sporophytes of *M. pyrifera* were used. These sporophytes were grown from spores under controlled conditions (as described by [33]) until blades were 5 cm in length. After this period, the sporophytes were randomly separated into 3 Erlenmeyer flasks of 2 L with 16 individuals per treatment and were subsequently exposed for 7 days to (i) 0.22 µm filtered seawater (control condition), (ii) Cu + Cd, (iii) Cu + PAHs, (iv) Cd + PAHs, and (v) Cu + Cd + PAHs. The specific concentration of each pollutant for the in vitro exposure treatments were according to the EC_20_ (20% effect concentration) values previously assessed for sporophyte development in our laboratory. In this previous study, independent Cu, Cd, and PAH (in vitro) treatments were carried out, where juvenile sporophytes were exposed during 7 days to the nominal concentrations of Cu, Cd and PAHs in the ranges 5–200 µg L^−1^, 5–200 µg L^−1^, and 0.05–100 µg L^−1^, respectively, determined on previous reports of Cu, Cd, and PAHs concentrations tested in seaweeds and other organisms from polluted environments [2,6,34]; then, EC_20_ values for Cu, Cd, and PAHs were determined using the ED function of the DRC package in the statistical environment R (R Development Core Team 2020). Specifically, the nominal effective concentrations EC_20_ were Cu = 190 μg L^−1^, Cd = 155 μg L^−1^, and PAHs = 0.65 μg L^−1^. The stock solutions of each metal and the PAHs mixture were prepared from analytical reagent-grade copper chloride (CuCl_2_, Titrisol^®^, Merck, Darmstadt, Germany), cadmium chloride (CdCl_2_ Titrisol^®^, Merck, Darmstadt, Germany), and the reagent QTM PAH Mix (Supelco, Bellafonte, PA, USA). The PAH mixture used contained 16 compounds considered PAH-priority pollutants by the US EPA. All the glass and plastic materials used in the experiments were washed and rinsed according to [2] for metals, and US EPA Method 610 for PAHs. The culture conditions were as follows: 14 °C, continued aeration provided by bubbling through air stones, a photoperiod of 12 h light and 12 h dark, and an irradiance of 30–40 µmol photons m^−2^ s^−1^.

### 2.2. Growth and Morphometry

After 7 days of exposure to experimental treatments, all *M. pyrifera* sporophytes were transferred to the sea (Quintay Bay, Valparaíso Region, Chile (33°11′29″ S; 71°42′16″ W)) and cultured for 90 days during the spring and summer seasons (from September to December). Quintay is characterized by a sandy bottom and rocky boulders, with an average depth of 8 m. Sporophytes from each treatment group were attached to a polypropylene rope (5 cm long and 3 mm Ø) and then tied to a 7 mm Ø nylon rope in a long-line culture system.

To evaluate the effects of the treatments on the growth and morphometric characters, 5 sporophytes were sampled from each treatment at 30, 60, and 90 days of culture. Specifically, for each treatment, the total wet weight was determined using a digital balance (±0.1 g) (Kern, Balingen, Germany), the number of blades were counted, and the width and height of the holdfast and blade were measured. 

### 2.3. Community Structure 

To determine the community structure associated with *M. pyrifera*, 5 sporophytes were harvested for each treatment at 90 days of cultivation. Subsequently, the epibiontic organisms were manually collected and kept in plastic containers with 70% ethanol–methanol 1:1 at 4 °C until identification using a stereomicroscope (Nikon SMZ1270, Tokyo, Japan). Additionally, the organisms were classified according to the thallus section where they were found, i.e., holdfast or blade. Finally, the organisms were identified at the lowest possible taxonomic level using specialized literature. The species richness, Shannon–Wiener diversity (H’), and Simpson equitability (D’) indices were calculated from the abundance data, obtaining proportional abundance within the samples. The Pielou index (J’), which measures the proportion of the diversity observed in relation to the expected maximum, allowed us to observe the existence of dominant species in the samples. To obtain the similarity in species composition between the treatments, the Jaccard coefficient index (IJ) was calculated using species richness according to [7]. All community descriptors were calculated with the R software (R Development Core Team 2020), using the “BiodiversityR” and “vegan” packages, and the resulting values were classified as high, medium, or low according to the literature [7,35,36]. 

### 2.4. Statistical Analyses

To evaluate the effects of the treatments and the time of culture on the morphological characteristics of kelps, a general linear model (GLM) analysis was employed, where morphology was considered a dependent variable, and treatments and time were considered independent variables.

To evaluate the differences in the flora and fauna associated with *M. pyrifera* holdfasts and blades, Dunnett’s analysis was carried out to assess significant differences in the community descriptors between treatments. For this, each community descriptor was used as the dependent variable, while the treatments were used as independent variables. Finally, for the Jaccard similarity coefficient, a dendrogram was generated using the “abe4” package from R software.

## 3. Results

### 3.1. Growth and Morphometry of M. pyrifera

**Figure 1:**
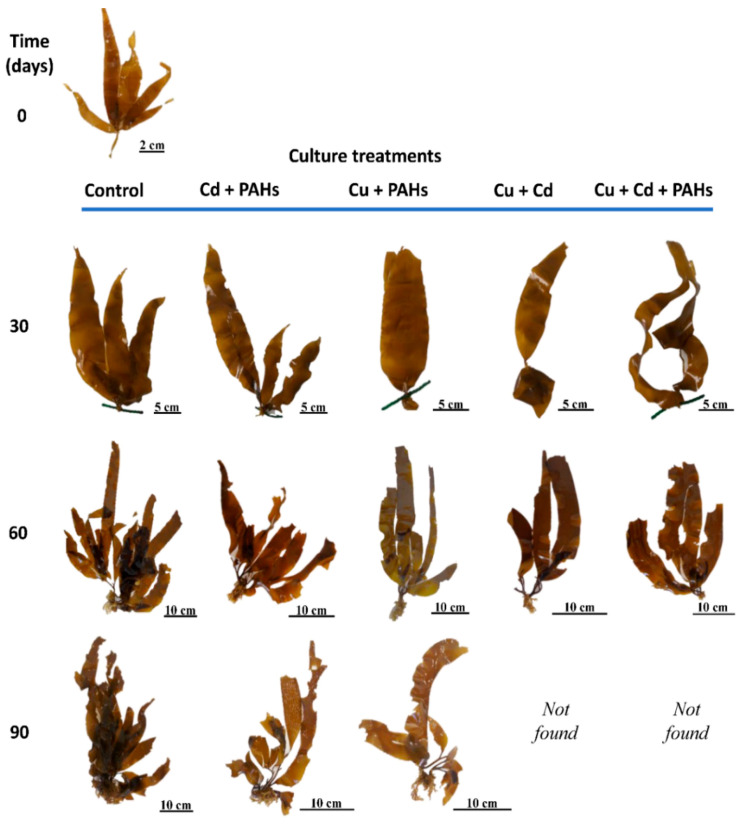
Representative images of *Macrocystis pyrifera* sporophyte from the control and from the heavy metal + PAHs treatments throughout the experimental period. Individuals from the treatments Cu + Cd and Cu + Cd + PAHs were not found at 90 days of culture.

**Figure 2:**
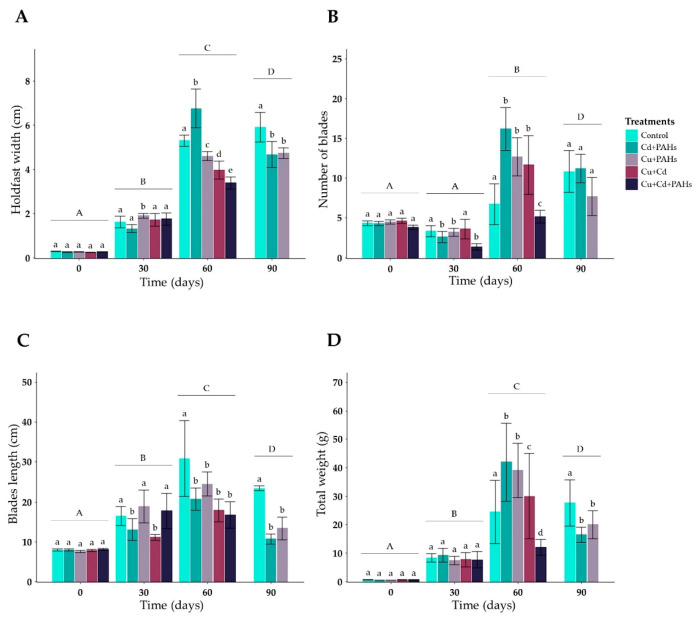
Morphometry of *Macrocystis pyrifera:* (**A**) holdfast width, (**B**) the number of blades, (**C**) blade length, and (**D**) total weight. *n* = 5 ± standard error. The significant differences between the treatments are shown with small letters, with capital letters indicating the differences between the experimental times (*p* < 0.05).

A constant increase was registered in all morphological features in the control individuals, where the highest values recorded were principally on day 90 of culture (Figure 1 and Figure 2 and Figure S1). On the other hand, the algae exposed to Cu + Cd and Cu + Cd + PAHs were not present in the culture line after 90 days of sea cultivation, strongly suggesting that the death of the individuals was due to the longer period of exposure to these toxicant mixtures. Additionally, these individuals presented the lowest values in all morphometric traits, especially those from the Cu + Cd + PAHs treatment (Figure 1 and Figure 2 and Figure S1). The results of the statistical analysis of the data are shown in Tables S1 and S2.

The holdfast width of the control individuals increased 9.8 times after 60 days of culture, reaching 5.3 ± 0.2 cm (Figure 2A). At this time, the thinnest holdfasts were recorded in the treatments Cu + Cd and Cu + Cd + PAHs, which had the lowest width of the period with 4.6 ± 0.6 cm and 3.4 ± 0.3 cm, respectively. After 90 days, the holdfast width from the control individuals reached 6.7 cm on average, increasing 20 times in relation to the initial time (Figure 2A). The holdfast height (Figure S1A) of the control individuals (5.3 ± 0.5 cm) increased 6.8 times after 60 days of culture. At this time, the individuals from the Cu + Cd + PAHs treatment presented the lowest average values (4.1 ± 0.5 cm) (Figure S1A).

In relation to the number of blades, the individuals from the Cd + PAHs treatment registered the largest values after 60 days of exposure compared to the initial cultivation time (4.3 ± 0.3 blades vs. 16.2 ± 2.7 blades) (Figure 2B). On the contrary, the lowest number of blades was registered in the individuals exposed to the treatments Cu + Cd and Cu + Cd + PAHs (11.7 ± 3.7 blades and 5.2 ± 0.8 blades, respectively) (Figure 2B). After 90 days, no statistical differences (*p* > 0.05) were registered between the individuals that survived from the three treatments. Specifically, at this time, the individuals presented an average of 10.8 ± 2.6 blades (control), 11.3 ± 1.8 blades (Cd + PAHs), and 7.7 ± 2.4 blades (Cu + PAHs).

A constant increase in the length of the blades was observed over time, with the control group presenting the highest values (Figure 2C), which increased three times by day 60 of the culture in comparison with the initial time (Figure 2C). Additionally, the length of the blades in the control treatment was 1.4 and 1.2 times greater in relation to the Cu + Cd + PAH and Cu + Cd treatments, respectively. On day 90, the control registered values were 1.6 and 2 times higher than the Cd + PAH and Cu + PAH treatments, respectively (Figure 2C).

Regarding the total weight, significant differences were observed throughout the cultivation time. The lowest values were observed after 60 days in the individuals exposed to the Cu + Cd + PAHs treatment (12.0 ± 2.8 g), and these were 28% lower compared to the Cd+PAHs (42.0 ± 13.7 g) and 48% lower compared to the control group (24.5 ± 11.1 g) (Figure 2D). After 90 days, the highest weight values were recorded for the control group; these values were 50 times higher compared to the initial time (27.7 ± 8.1 g) and 1.7 and 1.3 times higher than the Cd + PAHs (16.5 ± 2.7 g) and Cu + PAHs (20.0 ± 4.9 g), respectively (Figure 2D). 

The holdfast weight showed a continuous increase during the experimental period, registering the first significant differences (*p* < 0.05) on day 60 of the culture, where the lowest values were recorded in the individuals exposed to the Cu + Cd + PAHs (2.4 ± 0.4 g) (Figure S1B). On day 90 of the culture, the highest holdfast weight values were recorded in the control group (9.7 ± 2.8 g); they were 2.4 and 2.9 times higher than the Cd + PAHs (4.0 ± 0.6 g) and Cu + PAHs (3.4 ± 1.4 g), respectively (Figure S1B). Finally, the weight of the blades presented the highest values on day 60, and the low values were registered in individuals from the Cu + Cd + PAHs treatment (10 ± 2.6 g) (Figure S1C).

### 3.2. Community Structure Associated with M. pyrifera

**Figure 3:**
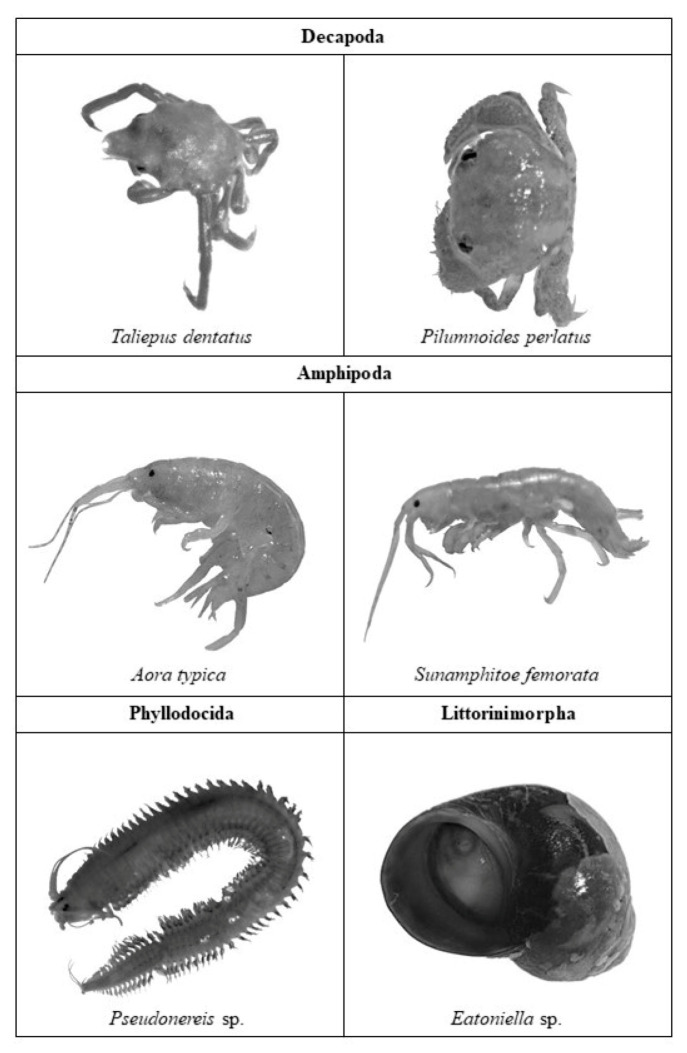
Representative images of the identified species associated with *Macrocystis pyrifera* thallus.

A total of five phyla and 16 species were associated with *M. pyrifera* thalli; 12 species were found in the control, 10 in the Cd + PAHs treatment, and 8 in the Cu + PAHs treatment (Table 1 and Figure 3). The most representative species considering all parts of the kelp (blades plus holdfast) corresponded to *Aora typica* and *Membranipora membranacea* for all treatments, with 52% and 25.7% from control individuals, 19% and 28.6% from Cd + PAHs individuals, and 25.5% and 43.1% from Cu + PAHs individuals, respectively.

In the biodiversity analysis of *M. pyrifera*, it was found that, for all the organisms present in the holdfast, the most abundant corresponded to amphipods (72%) and polychaetes (18.3%) considering all treatments (control = 83.7% and 8.7%, Cd + PAHs = 35% and 45%, and Cu + PAHs = 52.6% and 36.8%), the Gastropoda class (0.8%) being less represented. The total organisms found in the blades reveal a greater abundance of those from the Bryozoa phylum, with *M. membranacea* (61%) considering all treatments (control = 59.2%, Cd + PAHs = 54.5%, and Cu + PAHs = 69%), while the less abundant organism corresponded to the Polychaeta class (6.4%) in all treatments (control = 4.2%, Cd + PAHs = 13.6%, and Cu + PAHs = 6.3%).

**Figure 4:**
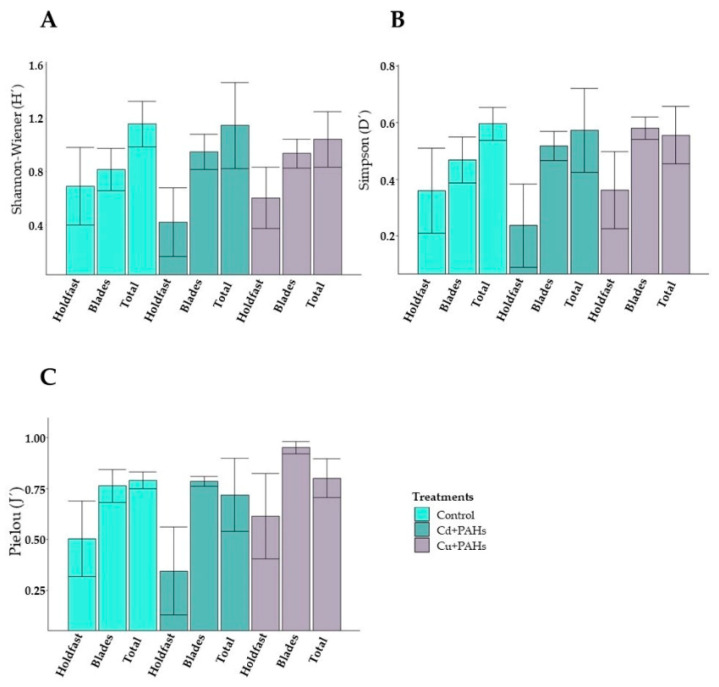
Community index determined in the holdfast, the blades, and the total (complete) thallus of *Macrocystis pyrifera* subject to different treatments (control, Cd + PAHs, and Cu + PAHs). Shannon–Wiener (**A**), Simpson (**B**), and Pielou (**C**) indexes. *n* = 5 ± standard error.

No significant differences were found in the Shannon–Wiener, Simpson, and Pielou indices between different parts of the kelp or between treatments (*p* > 0.05 in all cases, according to Dunnett´s test) (Figure 4). Nonetheless, the GLM analysis evidenced that these indices were mainly influenced by the localization of the organisms along the thallus (Figure 4 and Table S3). Particularly, the Shannon–Wiener index ranged between 0.42 and 1.16 (Figure 4A), indicating a low species diversity. The holdfast of *M. pyrifera* control presented the lowest species diversity (0.57 ± 0.08); on the contrary, a high diversity (1.21 ± 0.12) was observed when considering all thallus (blades plus holdfast) (Figure 4A). 

Despite non-statistically significant results, the Simpson (Figure 4B) and Pielou indices (Figure 4C) revealed clear differences between the treatments. In terms of the holdfast, a low species dominance was observed for all treatments (control = 0.38 ± 0.15; Cd + PAHs = 0.23 ± 0.15; Cu + PAHs = 0.36 ± 0.14) (Figure 4B). The holdfasts from the Cd + PAHs treatment presented low species evenness (0.34 ± 0.22) (Figure 4C), contrary to the control and the Cu + PAHs treatment, which showed an intermediate evenness (0.5 ± 0.19 and 0.61 ± 0.21, respectively). On the other hand, the control and Cd + PAHs treatments displayed a low species dominance (0.46 ± 0.08 and 0.51 ± 0.1, respectively) on the blades, in contrast to the Cu + PAHs, which had an intermediate dominance (0.58 ± 0.04) (Figure 4B). Considering all parts of the kelp, an intermediate species dominance was found in the control (0.6 ± 0.06) and Cu + PAHs treatments (0.56 ± 0.1), whereas a high dominance was found in the Cd + PAHs treatment (0.62 ± 0.05) (Figure 4B). All treatments exhibited high species evenness, taking into consideration all parts of the kelp (control = 0.79 ± 0.04; Cd + PAHs = 0.72 ± 0.2; Cu + PAHs = 0.8 ± 0.1), revealed by the Pielou index (Figure 4C). 

**Figure 5:**
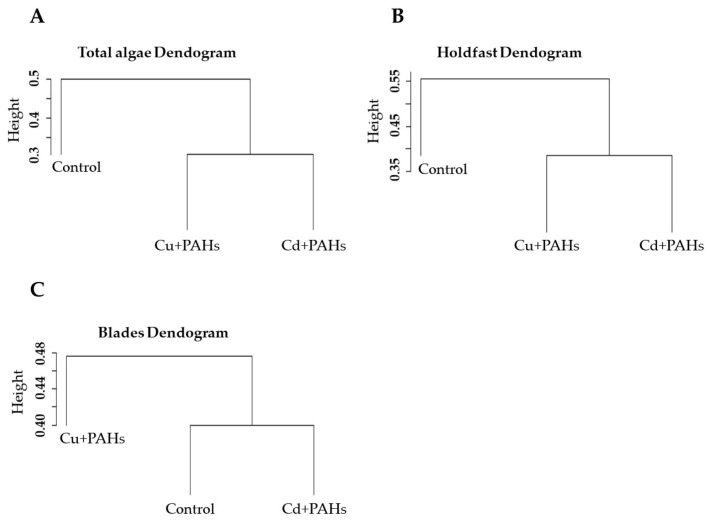
Dendrogram of the Jaccard similarity coefficient for the fauna associated with *Macrocystis pyrifera*. (**A**) Total algae, (**B**) holdfasts, and (**C**) blades. The values are in a range from 0 to 1.

The dendrogram based on the Jaccard index showed a clear separation between the control and the groups exposed to binary mixtures of toxics (Figure 5A). A similar pattern was observed for the holdfast (Figure 5B). Nonetheless, on the blades, the first group was composed of individuals exposed to the Cd + PAHs binary mixture and the control, and the second group included algae from the Cu + PAHs treatment (Figure 5C).

## 4. Discussion

The development of *M. pyrifera* sporophytes were negatively affected by a short pulse of exposure to mixtures of contaminants (heavy metals and PAHs), with visible morphological and development differences. The greatest negative effects were observed in the individuals exposed to Cu + Cd and Cu + Cd + PAHs regarding the weight, height, and width of the holdfast, the number of blades, and the length and weight of the blades. On the other hand, the role of *M. pyrifera* as an ecosystem engineer was also affected by pollutants, which modified the structure of the associated fauna and reduced the abundance of grazers. Concerning the community descriptors, although there were clear differences in some indices, no statistically significant differences were registered between the treatments. However, the Jaccard index showed groupings that suggest disparity and distinctive effects of the toxic mixtures on the community structure. In this way, this work evidenced the negative effects caused by the combination of heavy metals and PAHs on the development of *M. pyrifera* and its ecological role in the subtidal ecosystem.

The field experiments showed that, on day 90 of the culture post-exposure, the Cu + Cd and Cu + Cd + PAHs treatments exerted a negative effect on *M. pyrifera*. It has been widely shown that Cu generates reactive oxygen species (ROS) that damage the cellular components of seaweeds capable of inhibiting photosynthesis, reducing both their pigments and growth [37]. On the other hand, similar effects have been described in seaweeds exposed to Cd, the most widely described being the inhibition of photosynthesis, the degradation of pigments, and the denaturation of fatty acids by ROS [38,39,40]. The equitoxic mixture of Cu + Cd has been described as showing an antagonistic interaction effect, since these metals compete for active sites in the cell, but also as showing additive or synergistic effects, depending on the life cycle stage or the species being evaluated, and depending on the specific metabolic regulation of different metals at the cellular level. In the case of *M. pyrifera*, it has been observed that, in early stages of development, the binary mixture of these heavy metals generates antagonistic effects [19]. In addition, in the microalga *Chlorella* sp. at the cellular level, Cd and Cu co-exposure increases Cu (extracellularly and intracellularly) but inhibits Cd uptake, compared to each metal present by itself [40]. The same trend has been verified in a previous study in the kelp *Lessonia berteroana*, where, in Cu-polluted coastal areas, compared to reference sites without co-exposure with Cd, the Cd concentrations in algal tissues were much lower, and Cu levels, on the contrary, were much higher [41]. However, the results obtained in this work do not agree with those findings, since the Cu + Cd mixture was one of the mixtures that generated the greatest negative effects on the development of *M. pyrifera*; we therefore suggest a significant incorporation/effects of both heavy metals with the kelp tissue. 

It has been previously described that PAHs exposure generates ROS and decreases the fluidity and conductivity of the membranes [42,43]. In addition, synergistic effects have been determined for exposure to heavy metals and PAH mixtures, because both affect the fluidity of the membrane and its ionic balance, which positively influence the entry of heavy metals into the cell [44]. This agrees with the results obtained in this work, where the Cu + Cd + PAHs mixture generated the greatest effects on the development of *M. pyrifera*. In fact, the individuals of *M. pyrifera* exposed to this mixture of pollutants showed the greatest negative effects on their development (Cd + Cu and Cd + Cu + PAHs) and did not survive after day 60, with no thalli of this treatment found on day 90 of the culture. One possible explanation for this is that exposure to the Cu + Cd + PAHs caused the death of the *M. pyrifera* individuals due its high toxicity and synergistic interaction. Similar results were determined in the *Lessonia spicata* kelp using seawater (as a culture medium) from sites highly impacted by heavy metal and PAHs pollution [2]. Particularly, in vitro experiments with field-collected seawater showed that sites with high anthropogenic contamination induce sublethal effects on early developmental stages and, consequently, on seaweed populations, which in turn can also have long-term negative impacts on the community structure.

Regarding the fauna associated with *M. pyrifera*, 16 species were found (Table 1), which is considered low compared to what has been reported in natural beds [45,46]. Among the species found, the most abundant corresponded to the amphipod *A. typica*, followed by the bryozoan *M. membranacea*. The greatest abundance of *A. typica* is found in the holdfast together with *P. australis*, while *M. membranacea* is found exclusively in the blades. This agrees with what was previously described by [47,48], which indicates that the life cycle of these organisms develops mainly on the fronds of their algae host, reaching its highest abundance in the spring and summer periods. On the other hand, in the holdfast, the presence of sedentary tubicolous (Terebellidae) and mobile polychaetes was mainly recorded, which are mainly detritivores (Phyllodocidae, Pseudonereis, and *P. australis*) [49], as well as a species of crab of the genus *Taliepus* (*T. dentatus*), which have been described as common inhabitants of the kelp forest [50] and as important predators of *M. pyrifera* [51].

The absence of endophytic and epiphytic algae could be explained by certain traits of brown algae, such as *M. pyrifera*, which are able to chelate and exude metals through the generation of mucilage under contamination exposure. This allows them to generate high contaminant concentrations on their surface, inhibiting the settlement of other algal species [52]. Indeed, recent studies have proven the negative effects on the settlement and germination of algae exposed to seawater from zones polluted with these toxics [2]. 

The community indices showed low diversity and abundance values, in comparison to other reports. These differences could be explained mostly by the size of the algae, since Miller et al. [29] showed that there is a direct relationship between size of the algae and the number of associated species. Although there were no significant statistical differences in the community indices of the control algae compared with those exposed to the binary mixtures of pollutants (Cd + PAHs and Cu + PAHs), the dendrogram for the holdfast community based on the Jaccard index generated two groups, separating the algae of the control from that of these two mixtures—treatments that survived until day 90 of exposure. This disparity can be explained by the species present. In the case of the total algae of the control treatment, a greater abundance of organisms such as the amphipod *A. typica* and the bryozoan *M. membranacea* were registered, as were some exclusive species (*Amphipoda* sp. 2 and 3, *Pseudonereis* sp., and *Eatoniella* sp.) (Table 1). Likewise, in the case of the holdfast, a greater abundance of total organisms and a high abundance of *A. typica* were found in the control treatment, in addition to the presence of two exclusive species (*Pseudonereis* sp. and *Eatoniella* sp.) (Table 1). These differences could be the main reason that the Jaccard index separates the control treatment from algae exposed to contaminants. On the other hand, in the case of the organisms found in the blades, the dendrogram grouped the control algae with those exposed to Cd + PAHs. This can be explained because the Cu + PAHs treatment presents a lower richness and abundance, along with an accidental species (Foraminifera) (Table 1). Another difference is the number of organisms that feed on *M. pyrifera*, which present a higher abundance in the control group compared to other treatments. This is mainly due to the state of the algae, since it has been shown that pollutants change the palatability of algae, reducing their consumption by predators [53], preferring algae that have not been exposed to a secondary mixture of pollutants. 

## 5. Conclusions

This work evidenced the damage produced by a short pollution pulse in early *M. pyrifera* sporophytes, affecting its development and consequently its role as an ecosystem engineer. More specifically, this work revealed a decrease in the size and number of blades, a smaller holdfast, and even the death of *M. pyrifera* sporophytes exposed to a binary mixture of heavy metals and a tertiary mixture of pollutants, especially those exposed for a longer period of time. In addition, a low richness, diversity, and evenness was revealed in the sporophytes treated with toxic mixtures, compared to those of the control, likely due to a lower number of species associated with *M. pyrifera* holdfasts with smaller sizes and fewer blades. These alterations of *M. pyrifera* are likely to have important impacts on the community structure, which could also affect superior organisms in the trophic web such as mobile predators. 

## Figures and Tables

**Table 1:** Macroinvertebrates found in the holdfast and blades of *Macrocystis pyrifera* and their respective abundances, for algae exposed to a mixture of contaminants and the control group (without toxic exposure). * Indicate species that feed on *M. pyrifera*.

Phylum/Class	Taxa	Treatments					
		Control		Cd + PAHs		Cu +PAHs	
		Holdfast	Blades	Holdfast	Blades	Holdfast	Blades
Arthropoda							
Malacostraca	*Aora typical* *	77	9	7	1	10	3
	*Sunamphitoe femorata* *	-	4	-	1	-	1
	*Pilumnoides perlatus*	-	1	-	1	-	-
	*Taliepus dentatus* *	4	7	3	2	2	1
	*Erichthonius* sp.	2	-	1	-	-	-
	Amphipoda sp.1	-	-	-	2	-	2
	Amphipoda sp.2	-	3	-	-	-	-
	Amphipoda sp.3	-	2	-	-	-	-
Annelida							
Polychaeta	*Platynereis australis*	6	3	6	1	5	2
	*Pseudonereis* sp.	1	-	-	-	-	-
	Phyllodocidae	-	-	-	2	-	-
	Terebellidae	1	-	3	-	2	-
Mollusca							
Gastropoda	*Eatoniella* sp.*	1	-	-	-	-	-
Bryozoa							
Gymnolaemata	*Membranipora* *membranacea*	-	42	-	12	-	22
Foraminifera	Foraminifera	-	-	-	-	-	1
Total individuals		92	71	20	22	19	32
Total identities		7	8	5	8	4	7

## Data Availability

Derived data supporting the findings of this study are available from the corresponding authors (L. Contreras-Porcia and C. Bulboa) on request.

## Supplementary Materials

The following are available online at https://www.mdpi.com/article/10.3390/toxics9080190/s1, Figure S1: Morphometry of *M. pyrifera*: holdfast height (A), holdfast weight (B), and blade weight (C), Table S1: Analysis of variance (ANOVA) of a generalized linear model (GLM) of morphometric measurements (blade length, the number of blades, and blade weight) at 90 days of cultivation in response to combined contaminants (Cu + Cd, Cu + PAHs, Cd + PAHs, and Cu + Cd + PAHs). Table S2: Analysis of variance (ANOVA) of a generalized linear model (GLM) on morphometric measurements (holdfast width, height, weight, and total weight) at 90 days of cultivation in response to combined contaminants (Cu + Cd, Cu + PAHs, Cd + PAHs, and Cu + Cd + PAHs). Table S3: Analysis of variance (ANOVA) of a generalized linear model (GLM) of the community descriptors (Shannon–Wiener, Simpson, and Pielou) at 90 days of cultivation in response to combined contaminants (Cu + Cd, Cu + PAHs, Cd + PAHs, and Cu + Cd + PAHs).
