# Supplementary material for: Negative Consequences on the Growth, Morphometry, and Community Structure of the Kelp Macrocystis pyrifera (Phaeophyceae, Ochrophyta) by a Short Pollution Pulse of Heavy Metals and PAHs
Source: Toxics. 2021 Aug 18;9(8):190. doi: 10.3390/toxics9080190 (PMC8402373; doi:10.3390/toxics9080190)
Supplement: Supplementary file 1 [file toxics-09-00190-s001.zip › toxics-1329503-supplementary.pdf]

toxics

MDPI

Supplementary Materials: Negative Consequences on the Growth, Morphometry, and Community Structure of the Kelp *Macrocystis pyrifera* (Phaeophyceae, Ochrophyta) by A Short Pollution Pulse of Heavy Metals and PAHs

Roddy Jara-Yáñez, Andrés Meynard, Gladys Acosta, Nicolás Latorre-Padilla, Carolina Oyarzo-Miranda, Francisco Castañeda, Florentina Piña, Jorge Rivas, Cristian Bulboa and Loretto Contreras-Porcia

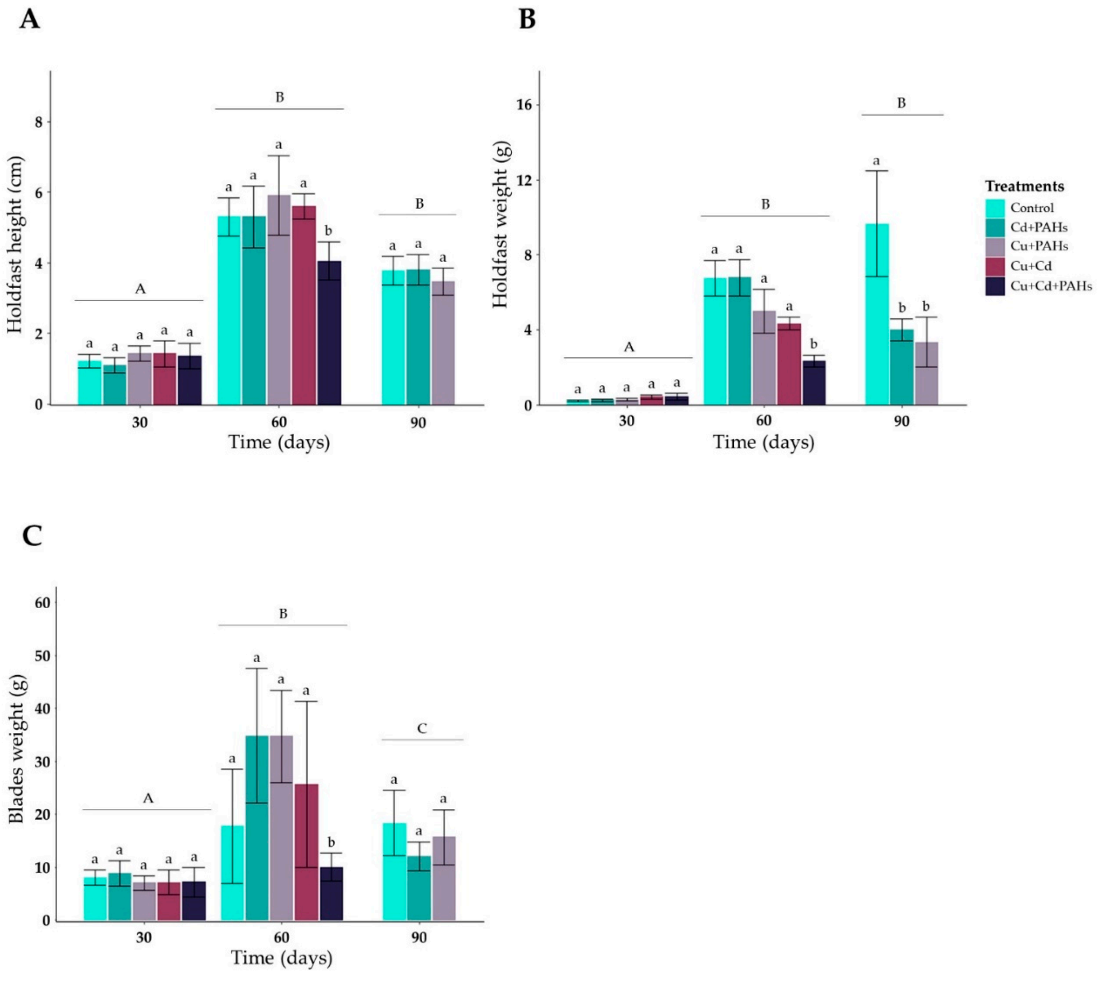

Figure S1. Morphometry of *M. pyrifera*: holdfast height (**A**), holdfast weight (**B**), and blade weight (**C**). $n = 5 \pm$ standard error. The significant differences between the treatments are shown with small letters, and differences between the times of culture are shown with capital letters ($p < 0.05$).

Table S1. Analysis of variance (ANOVA) of a generalized linear model (GLM) of morphometric measurements (blade length, the number of blades, and blade weight) at 90 days of cultivation in response to combined contaminants (Cu + Cd, Cu + PAHs, Cd + PAHs, and Cu + Cd + PAHs). * Corresponds to a statistical interaction between groups.

Predictors	Blades Length			No. of Blades			Blades Weight		
	Inc. Rate[1]	Ratios CI	*p*	Inc. Rate	Ratios CI	*p*	Inc. Rate	Ratios CI	*p*
(Intercept)	8.02	7.28–8.81	**<0.001**	4.28	3.72–4.89	**<0.001**	8.88	3.24–18.9	**<0.001**
Control	0.99	0.86–1.14	0.885	1.01	0.83–1.23	0.898	0.90	0.27–3.14	0.868
Cu + Cd	0.98	0.85–1.12	0.769	1.08	0.89–1.31	0.433	0.81	0.20–2.98	0.746
Cu + Cd + PAHs	1.01	0.89–1.16	0.832	0.90	0.73–1.10	0.301	0.81	0.21–2.99	0.746
Cu + PAHs	0.94	0.82–1.08	0.402	1.05	0.86–1.28	0.616	0.79	0.20–2.94	0.725
Time 30	1.84	1.42–2.35	**<0.001**	0.61	0.32–1.05	0.103	-	-	-
Time 60	3.06	2.49–3.73	**<0.001**	3.79	2.87–4.93	**<0.001**	3.92	1.60–11.6	**0.008**
Time 90	1.85	1.39–2.42	**<0.001**	2.63	1.84–3.65	**<0.001**	1.35	0.40–4.70	0.625
Control*Time 30	1.07	0.76–1.50	0.701	1.27	0.59–2.81	0.550	-	-	-
Cu + Cd*Time 30	1.07	0.75–1.52	0.711	1.28	0.59–2.88	0.536	-	-	-
Cu + Cd + PAHs* Time 30	1.56	1.13–2.17	**0.007**	0.60	0.21–1.59	0.318	-	-	-
Cu + PAHs * Time 30	1.20	0.85–1.70	0.307	1.17	0.52–2.67	0.703	-	-	-
Control*Time 60	0.87	0.64–0.19	0.398	0.41	0.24–0.67	**0.001**	0.56	0.13–2.41	0.443
Cu + Cd*Time 60	0.72	0.50–1.02	0.070	0.67	0.41–1.05	0.088	0.91	0.20–4.36	0.909
Cu + Cd + PAHs* Time 60	0.60	0.44–0.81	**0.001**	0.36	0.22–0.57	<**0.001**	0.36	0.07–1.74	0.198
Cu+PAHs * Time 60	0.99	0.71–1.37	0.955	0.74	0.47–1.16	0.204	1.26	0.28–5.88	0.762
Control*Time 90	1.45	1.03–2.04	**0.034**	0.95	0.61–1.50	0.826	1.69	0.35–8.41	0.512
Cu+PAHs * Time 90	0.93	0.60–1.42	0.725	0.65	0.36–1.13	0.138	1.65	0.28–10.0	0.579

[1]Incidence Rate.

Table S2. Analysis of variance (ANOVA) of a generalized linear model (GLM) on morphometric measurements (holdfast width, height, weight, and total weight) at 90 days of cultivation in response to combined contaminants (Cu+Cd, Cu+PAHs, Cd+PAHs, and Cu+Cd+PAHs). * Corresponds to a statistical interaction between groups.

Predictors	Holdfast Width			Holdfast Height			Holdfast Weight			Total Weight[2]		
	Inc. Rate[1]	Ratios CI	*p*	Inc. Rate	Ratios CI	*p*	Inc. Rate	Ratios CI	*p*	Inc. Rate	Ratios CI	*p*
(Intercept)	0.26	0.22–0.29	**<0.001**	1.10	0.65–1.72	0.699	0.24	0.03–0.87	0.095	0.53	0.33–0.79	**0.004**
Control	1.14	0.95–1.38	0.169	1.11	0.59–2.12	0.757	0.90	0.08–11.7	0.930	1.05	0.57–1.94	0.872
Cu+Cd	0.94	0.77–1.15	0.550	1.29	0.68–2.48	0.437	1.75	0.23–20.3	0.596	1.06	0.58–1.95	0.850
Cu+Cd+PAHs	1.06	0.87–1.28	0.578	1.24	0.65–2.39	0.522	1.83	0.25–21.1	0.563	1.17	0.65–2.15	0.597
Cu+PAHs	1.02	0.84–1.24	0.846	1.29	0.68–2.48	0.437	1.17	0.11–14.8	0.893	0.98	0.52–1.84	0.947
Time 30	5.13	4.02–6.50	**<0.001**		-	-	-	-	-	17.4	10.1–30.6	**<0.001**
Time 60	26.2	22.3–30.9	**<0.001**	4.82	2.92–8.45	**<0.001**	28.3	7.45–269	**<0.001**	79.5	51.5–129	**<0.001**
Time 90	18.1	15.1–21.7	**<0.001**	3.45	2.02–6.22	**<0.001**	16.6	4.16–161	**0.002**	31.2	18.9–53.4	**<0.001**

Control*Time 30	1.07	0.78–1.48	0.670	-	-	-	-	-	-	0.86	0.40–1.86	0.701
Cu+Cd*Time 30	1.38	1.00–1.93	0.054		-	-	-	-	-	0.79	0.36–1.73	0.552
Cu+Cd+PAHs* Time 30	1.26	0.91–1.75	0.163	-	-	-	-	-	-	0.71	0.32–1.54	0.381
Cu+PAHs *Time 30	1.41	1.02–1.96	**0.038**	-	-	-	-	-	-	0.81	0.36–1.84	0.616
Control*Time 60	0.69	0.54–0.87	**0.002**	0.90	0.44–1.84	0.783	1.10	0.08–13.6	0.936	0.55	0.28–1.09	0.086
Cu+Cd*Time 60	0.62	0.48–0.81	**<0.001**	0.82	0.39–1.69	0.593	0.36	0.03–3.00	0.359	0.67	0.34–1.32	0.249
Cu+Cd+PAHs* Time 60	0.47	0.37–0.60	**<0.001**	0.62	0.30–1.27	0.196	0.19	0.02–1.49	0.127	0.24	0.12–0.48	**<0.001**
Cu+PAHs *Time 60	0.67	0.52–0.86	**0.002**	0.86	0.41–1.78	0.691	0.63	0.05–7.34	0.696	0.95	0.48–1.89	0.880
Control*Time 90	1.11	0.87–1.41	0.402	0.90	0.43–1.88	0.781	2.68	0.20–33.5	0.411	1.59	0.79–3.21	0.189
Cu+PAHs *Time 90	0.99	0.76–1.30	0.958	0.71	0.32–1.54	0.389	0.71	0.05–8.64	0.780	1.24	0.58–2.64	0.578

[1]Incidence Rate, [2]Holdfast plus blades weight

Table S3. Analysis of variance (ANOVA) of a generalized linear model (GLM) of the community descriptors (Shannon–Wiener, Simpson, and Pielou) at 90 days of cultivation in response to combined contaminants (Cu+Cd, Cu+PAHs, Cd+PAHs, and Cu+Cd+PAHs). Those that present statistical differences are highlighted.

Index	Condition	Deviance	Resid. Dev	F	*P*r(>F)
Diversity of Shannon–Wiener (H′)	Pollutant	0.032	19.459	0.047	0.954
	Treatment	2.725	16.734	3.954	**0.028**
	Pollutant:Treatment	0.437	16.297	0.317	0.865
Simpson Equitability (D′)	Pollutant	0.077	9.499	0.232	0.794
	Treatment	1.223	8.276	3.687	**0.035**
	Pollutant:Treatment	0.190	8.086	0.287	0.884
Pielou Index (J′)	Pollutant	0.395	11.509	1.028	0.368
	Treatment	1.541	9.968	4.012	**0.026**
	Pollutant:Treatment	0.210	9.758	0.274	0.893
